# Human Innate Lymphoid Cells: Their Functional and Cellular Interactions in Decidua

**DOI:** 10.3389/fimmu.2018.01897

**Published:** 2018-08-14

**Authors:** Paola Vacca, Chiara Vitale, Enrico Munari, Marco Antonio Cassatella, Maria Cristina Mingari, Lorenzo Moretta

**Affiliations:** ^1^Department of Immunology, IRCCS Bambino Gesù Children’s Hospital, Rome, Italy; ^2^Department of Experimental Medicine (DIMES), University of Genoa, Genoa, Italy; ^3^UOC Immunology, IRCCS Ospedale Policlinico San Martino Genova, Genoa, Italy; ^4^Department of Pathology, Sacro Cuore Don Calabria Hospital, Negrar, Italy; ^5^Department of Pathology AOUI, University of Verona, Verona, Italy; ^6^Department of Medicine, Section of General Pathology, University of Verona, Verona, Italy; ^7^Center of Excellence for Biomedical Research (CEBR), University of Genoa, Genoa, Italy

**Keywords:** innate lymphoid cell, innate immunity, natural killer cells, human pregnancy, neutrophils, stromal cells, inflammation, tolerance

## Abstract

Innate lymphoid cells (ILC) are developmentally related cell subsets that play a major role in innate defenses against pathogens, in lymphoid organogenesis and in tissue remodeling. The best characterized ILC are natural killer (NK) cells. They are detectable in decidua in the early phases of pregnancy. During the first trimester, NK cells represent up to 50% of decidua lymphocytes. Differently from peripheral blood (PB) NK cells, decidual NK (dNK) cells are poorly cytolytic, and, instead of IFNγ, they release cytokines/chemokines that induce neo-angiogenesis, tissue remodeling, and placentation. dNK interact with resident myeloid cells and participate in the induction of regulatory T cells that play a pivotal role in maintaining an efficient fetal–maternal tolerance. dNK cells may originate from CD34^+^ precursor cells present *in situ* and/or from immature NK cells already present in endometrial tissue and/or from PB NK cells migrated to decidua. In addition to NK cells, also ILC3 are present in human decidua during the first trimester. Decidual ILC3 include both natural cytotoxic receptor (NCR)^+^ and NCR^−^ cells, producing respectively IL-8/IL-22/GM-CSF and TNF/IL-17. NCR^+^ILC3 have been shown to establish physical and functional interactions with neutrophils that, in turn, produce factors that are crucial for pregnancy induction/maintenance and for promoting the early inflammatory phase, a fundamental process for a successful pregnancy. While NCR^+^ILC3 display a stable phenotype, most of NCR^−^ILC3 may acquire phenotypic and functional features of NCR^+^ILC3. In conclusion, both NK cells and ILC3 are present in human decidua and may establish functional interactions with immune and myeloid cells playing an important role both in innate defenses and in tissue building/remodeling/placentation during the early pregnancy. It is conceivable that altered numbers or function of these cells may play a role in pregnancy failure.

## Introduction

The fetus can be considered as a semi-allograft to the maternal host; therefore, pregnancy should include mechanisms to prevent allograft rejection (1–3). During the early phases of pregnancy, an appropriate balance between inflammation and tolerance is critical for a successful pregnancy (4, 5). Indeed, pro-inflammatory cytokines have been shown to contribute to tissue building/remodeling and neo-angiogenesis, thus favoring embryo implantation (6–8). The inflammatory phase is followed by a regulatory phase characterized by an increase in regulatory T cells (Tregs) that prevent an excessive inflammation and avoid fetal immuno-mediated rejection (9). Thus, relevant interactions among cells involved in immune response may occur at the fetal–maternal interface and play a fundamental role for a successful pregnancy.

## General Characteristics of Innate Lymphoid Cells (ILC) Subsets

Innate lymphoid cells are immune effector cells involved in host defenses against pathogens and tumors, in lymphoid organogenesis and in secondary lymphoid organ remodeling after birth. ILCs are tissue-resident cells mainly found at the mucosal surfaces of intestine (10), lungs (11), decidua (12), and skin (13, 14). Thanks to their strategic location, ILC are among the first immune cells to respond to pathogens. Recently, on the basis of their cytokine profile and transcription factors (TF), ILC have been classified into two main groups: cytotoxic- and helper-ILC (15–18). Natural killer (NK) cells, representing cytotoxic-ILC, are the first innate lymphoid cell population described, featuring the capacity of killing virus-infected or tumor cells and to release pro-inflammatory cytokines and chemokines. Human NK cell function is regulated by an array of inhibitory receptors, such as the HLA-I-specific killer immunoglobulin-like receptors and CD94/NKG2A, and by activating receptors, including natural cytotoxicity receptors (NCR, i.e., NKp46, NKp30, and NKp44), NKG2D, DNAM-1, and CD16 (19, 20). The other ILC are represented by “helper”-ILC that are further classified into three main subsets (ILC1/ILC2/ILC3) (21). ILC1 mainly produce IFNγ and provide defenses against intracellular bacteria and protozoa (22). In humans, two different subsets of ILC1 have been described in the intestine (23, 24). ILC2 mainly release type-2 cytokines, such as IL-5, IL-13, and IL-4 and contribute to type-2 immune responses. Finally, ILC3 are a heterogeneous subset, their signature cytokines are represented by IL-17 and IL-22 (25). ILC3 were first identified in the fetus and were originally defined lymphoid tissue inducer (LTi) cells because of their key role in driving lymphoid organogenesis. In particular, during embryogenesis, LTi cells interact with stromal cells and induce upregulation of adhesion molecules thus promoting the development of lymph node structure. After birth, ILC3 are mainly located in secondary lymphoid organs (SLO), tonsils, decidua, and intestinal lamina *propria* where they contribute to host defenses against extracellular pathogens and are defined as LTi-like cells. In humans, LTi/LTi-like cells are lineage (CD3/CD19/CD14/CD56)-negative and express CD127, CD117, retinoic acid receptor-related orphan receptor (ROR)-γt TF, and secrete primarily IL-17 and TNFα. A population of cells referred to as NCR + ILC3, sharing common features with both LTi-like cells and NK cells (type of cytokines production and NCR expression, respectively), has recently been identified in mucosal tissues and prevalently releases IL-22. ILC3-derived IL-22 acts on intestinal epithelial cells and induces not only production of antimicrobial peptides but also epithelial cell migration and wound healing. Moreover, ILC3 promote tissue repair and remodeling of SLO damaged by inflammatory processes. Conversely, ILC3 may also exert a pro-inflammatory role in intestinal inflammatory diseases.

All ILC subsets are developmentally related. Evidence in mice and humans indicates that NK cells and helper-ILC derive from a common ILC progenitor (CILP). As B and T lymphoid progenitors, the CILP derive from the common lymphoid progenitor. The acquisition of mature stages is dependent by different TF. Thus, NK cell differentiation involves Eomes, which regulates the expression of IFNγ and of the cytolytic machinery, while terminal differentiation of helper-ILC is regulated by other TF. In particular, ILC1 requires Tbet, ILC2 GATA3, and RORα, and ILC3 RORγt and AhR (26–29). Although, specific ILC3-committed precursors have been defined, a precise identification of a common ILC precursor in humans is still lacking. Moreover, it is still only partially understood which signals from the microenvironment are driving their differentiation. The low numbers of ILC3 that can be generated *in vitro* has so far hampered studies aimed to answer these questions. Moreover, limitations in cell numbers may be an obstacle for clinical application of ILC. Thus, the development of protocols allowing the generation of suitable numbers of given subsets of ILC for their use in adoptive cell therapy is required.

Along this line, it has become more evident that the fate of ILC determination and their stability is not set in stone, but that there is some plasticity between different ILC subsets, depending on various signaling, including cytokines and exposure to different tissue-specific microenvironments. This would indicate that microenvironmental conditions might drive this plasticity from an ILC subset to another (29). Accordingly, it is conceivable that also putative differentiated ILC may display intermediate phenotypic/functional characteristics (30, 31).

## ILC in Human Decidua and Their Interactions with Decidua Microenvironment

Innate immune cells are important components of the decidual microenvironment. In this tissue, the best characterized and most abundant ILCs are NK cells (1, 2, 32). Remarkably, while the function of peripheral blood (PB) NK cells is to defend the host against infections and tumors, thanks to their cytolytic activity and production of cytokines, such as IFN-γ and TNF, decidual NK (dNK) cells are characterized by a regulatory function (33). It has been shown that the NK cell function is greatly influenced by the microenvironment, including cytokines (34), chemokines, and cell-to-cell interactions. A paradigmatic example of how NK cell function may be regulated in tissues is provided by human dNK cells. They represent as much as 50–70% of decidual infiltrating lymphocytes during the first trimester of pregnancy, and are characterized by CD56^bright^CD16^−^KIR^+^CD9^+^ phenotype (2, 35). In spite of their high content of cytolytic granules, dNK cells are poorly cytotoxic and release very low amounts of IFNγ as compared to PB-NK cells (2, 36–39). On the other hand, dNK cells release peculiar cytokines and chemokines, such as vascular endothelial growth factor (VEGF), stromal derived factor-1 (SDF-1 also identified as CXCL12), and IFN-γ-inducing protein 10 (IP10 also known as CXCL10), that mediate neo-angiogenesis, tissue remodeling, and placentation (3). Similarly, to humans, also murine dNK cells are abundant during the early phases of pregnancy and display unique phenotypic and functional features (40–44). Several reports revealed that NK cells are present also in non-pregnant endometrial tissue and that their proportions may vary during the menstrual cycle. It has been shown that, besides NK cells, other ILC populations are present in human decidua during the early phases of pregnancy (12, 41) This finding supports the notion that ILC participate to defensive/tissue building processes necessary for the maintenance/success of pregnancy (45, 46). Notably, decidual tissues contain different RORγt^+^ ILC3 subsets displaying functional features similar to those previously described in other tissues. In particular, dILC3 not only express IL-22, but are also the main IL-8 producers, a functional activity previously assigned to dNK cells. Moreover, dILC3 have been shown to induce the expression of adhesion molecules (a functional activity referred to LTi-like cells) on decidual stromal cells (DSC), thus suggesting that, also dILC3, may play a role in tissue building/remodeling during the early phases of pregnancy.

## Origin of Decidual ILC

Given the relevant role of dNK cells in the maintenance of pregnancy, an important issue is to clarify their origin. NK cells are known to originate from CD34^+^ hematopoietic stem cell, as revealed by a number of studies both *in vitro* and *in vivo*. These precursors are present mainly in the bone marrow, and also in PB and cord blood. Although, it is possible that dNK cells may derive from PB-NK cells migrated in decidua, where they acquire unique functional features upon exposure to the decidual microenvironment (47, 48), they may also derive from CD34^+^ precursors detectable in decidua. In this context, previous studies described the presence of CD34^+^VEGFR^−^ precursors in decidual tissues and of immature NK cells in endometrial tissue (47–51). Both cell populations could undergo differentiation into dNK cells during pregnancy. Remarkably, dCD34^+^ cells are committed toward the NK cell lineage as revealed by experiments showing that they undergo rapid *in vitro* differentiation toward mature NK cells both in the presence and in the absence of cytokines, provided they are co-cultured with DSC (3, 52).

The issue of the developmental relationship between different ILC is a matter of debate. In particular, the relationship between NK cells and ILC3 is unclear in humans, because the phenotypic features of immature NK cells and ILC3 are partially overlapping. It was originally proposed that NCR^+^ILC3 represent an immature stage of NK cell development (53, 54). However, decidual ILC3, despite their extensive proliferative capacity, maintain their phenotypic and functional characteristic, while only a minor fraction could differentiate toward CD94^+^ NK cells even in the presence of IL-15. Similarly, to NCR^+^ILC3, also LTi-like cells virtually failed to generate NK cells. Thus, it is conceivable that the majority of NK cells present in decidual tissue may derive from PB-NK cells migrated to decidua, as well as from CD34^+^ cell precursors detectable *in situ*. On the other hand, our recent studies support the notion that a developmental relationship exists between the two ILC3 subsets present in decidua. Indeed, NCR^+^ILC3 (expressing CD56) could be derived from LTi-like CD56^−^ cells. In these *in vitro* experiments, only a small percentage of LTi-like cells retained their phenotypic characteristics (Lin^−^CD56^−^CD117^+^CD127^+^), while they acquired the Lin^−^CD56^+^CD117^+^CD127^+^CD94^−^NCR^+^ phenotype (12). It cannot be excluded that, similarly to NK cells, also ILC3 may derive from precursors present in decidua. Indeed, ILC3 are not present in PB, implying that they should develop *in situ* from a precursor. Interestingly, dCD34^+^ precursors are characterized by the expression of ID2 transcription factor, which is required for ILC development, suggesting that dCD34 cells may give rise not only to dNK cells but also to NCR^+^ILC3 and LTi-like cells (12).

Although NK cells belong to the innate immune system, different reports suggested that they may display adaptive-like properties. These adaptive features include clonal expansion and the generation of long-lived memory cells (55–58). In humans, “trained/memory” NK cells are characterized by the expression of HLA-class I-specific activating receptor NKG2C (59). The NKG2C^+^ NK cell subset undergo great expansion following human cytomegalovirus infection (55, 60). Several observations indicate that a major risk of a deficient placentation occurs in women that undergo first pregnancy (61, 62). These data suggest that, after the first pregnancy, the uterine microenvironment may acquire the ability to better sustain the early phase of placentation, including the inflammatory process. In this context, it has been shown recently that the dNK cell repertoire significantly differs in primigravidae as compared to multigravidae. Mulitgravid women display higher percentages of dNK cells expressing NKG2C and LILRB1, which also produce higher amounts of IFN-γ and VEGF as compared to dNK cells detectable in primigravid women. Precursors of these cells are present in the uterus between pregnancies and may become activated by the uterine microenvironment once a new pregnancy occurs. These cells could represent “trained” NK cells that would improve endometrial vascularization, angiogenesis, and maintenance of decidua through a more prompt and abundant secretion of functional VEGF and IFN-γ (63).

## Involvement of ILC in the Inflammatory and Tolerogenic Phases of Pregnancy

A successful pregnancy requires an early inflammatory phase that is necessary for successful implantation, while, subsequently, a regulatory/immunosuppressive phase should follow to prevent fetal rejection (5, 8, 64). Several studies highlighted the role of innate and adaptive immune cells in promoting either an inflammatory or an immunosuppressive environment. It is conceivable that functional activities thought to be exclusive of adaptive immunity, may actually be mediated also by ILC (65). Thus, not only uterine Th17 but also dILC releasing pro-angiogenetic factors are likely to play a role in the neo-angiogenesis and antimicrobial defenses during pregnancy.

### The Inflammatory Phase

**Figure 1:**
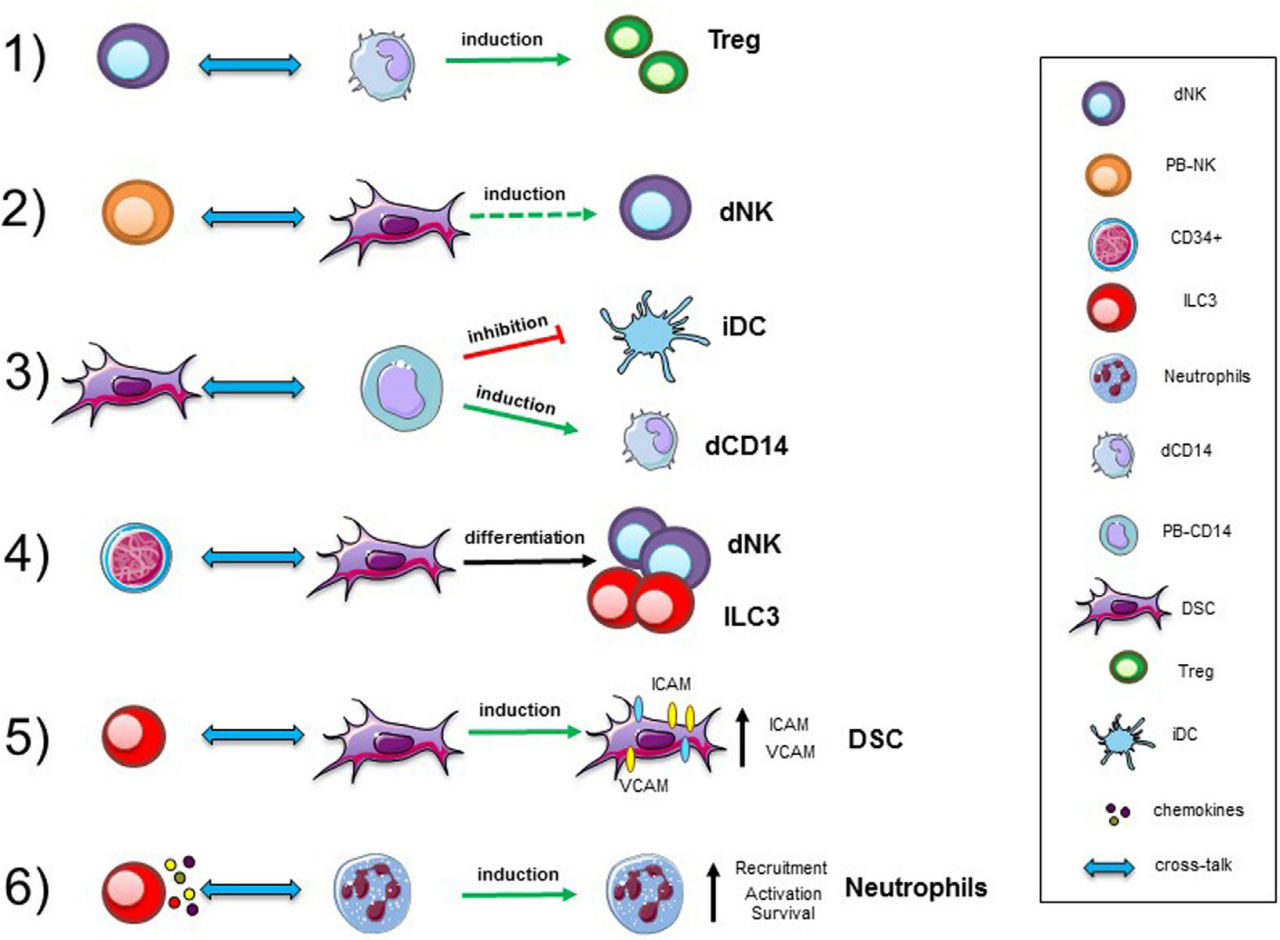
Cellular interactions in human decidua. Representative interactions between immune cells, precursors, and stromal cells occurring in decidual tissues during the first trimester of pregnancy. Abbreviations: dNK, decidual natural killer cells; PB-NK, peripheral blood NK cells; ILC, innate lymphoid cells; dCD14, decidual myeloid cells; PB-CD14, peripheral blood myeloid cells; DSC, decidual stromal cells; Treg, regulatory T cells; iDC, immature dendritic cells.

Different studies revealed the presence of neutrophils (N) with pro-angiogenetic capability in human decidua during the first and the second trimesters of pregnancy (66, 67). In particular, during the first trimester, N present in human decidual tissues are characterized by an “activated” phenotype, production of fibro/angiogenic factors and a prolonged survival. Previous studies reported that NCR^+^ILC3 could mediate neutrophil activation in spleen *via* GM-CSF (68). It has been shown that dILC3 could functionally interact with N in decidual tissues (Figure 1). In particular, it is conceivable that dN recruitment to decidua, their activation, and expression of peculiar immunoregulatory factors (HB-EGF and IL1ra) may be related to interaction with dILC3 (69, 70). Indeed, thanks to their ability to release IL8 and GM-CSF, dILC3 could promote neutrophil recruitment and activation, respectively. These observations support the notion that both ILC3 and N may play an important role in early inflammatory phase and, subsequently, in the induction of tolerance.

Recent studies revealed that also cytokines released by NK cells might induce neutrophil activation, expression of activation markers, and production of cytokines and angiogenic factors (71–73). In addition, under suitable experimental conditions, NK cells have been shown to induce neutrophil apoptosis (74). Reciprocally, N may inhibit NK cell proliferation, cytolytic activity, cytokine production, and survival *via* contact-dependent or -independent mechanisms (73). In agreement with the concept of NK/N interactions in decidua, it has been observed that dN localize in close proximity of CD56^+^ cells, suggesting a potential crosstalk with dNK cells. Thus, it is possible to speculate that also decidual N by interacting with dILC3 and/or dNK cells may contribute to the regulation of innate/adaptive immune responses occurring during pregnancy, by the release of soluble factors, or cell-to-cell contacts (67). These data shed new light on the cellular and molecular mechanisms involved in the initiation of an inflammatory response in the decidua.

### The Tolerogenic Phase

Since fetus is a semi-allograft, successful pregnancy should also include mechanisms capable of preventing allograft rejection. Indeed, while effective immune responses must be maintained in order to protect the mother from harmful pathogens, immune reaction against fetal antigens should also be avoided. dNK cells express normal levels of HLA-class I-specific inhibitory receptors. Notably, certain HLA-class I molecules are expressed by trophoblast and are involved in the regulation of trophoblast growth, differentiation, and invasion (75). The fact that dNK cells are unable to kill different target cells has been tentatively explained with the poor ability to form appropriate immunological synapses and/or the expression of the inhibitory form of 2B4 co-receptor (39, 76). In addition, the fact that dNK cells do not kill trophoblastic cells has also been explained with the expression of inhibitory NK receptors, such as CD94/NKG2A specific for HLA-E, recognizing HLA-G KIR2DL4 and KIR2DL1/2/3 specific for HLA-C, i.e., the HLA-class I molecules expressed by human trophoblast. Therefore, both the poor ability to kill of dNK cells and a number of fail-safe mechanism may be responsible for the inability of dNK cells to kill the invading trophoblast (7). Notably, also Tregs have been detected during early pregnancy and are thought to exert an important protective role when the maternal immune cells come into contact with fetal antigens expressed by invading trophoblast cells. Accordingly, studies in humans revealed the presence of Tregs in the PB during the early phases of normal pregnancy, while a low Treg numbers have been reported in cases of recurrent pregnancy loss (77). In this context, it should be stressed that different Treg subpopulations occur, including naturally occurring Tregs, which derive from the thymus, and adaptive Tregs, which develop in the periphery, The development of Tregs requires the transcription factor Forkhead box P3 which currently represents their most specific marker. The secretion of inhibitory cytokines and contact-dependent inhibition are two identified mechanisms of Treg-mediated suppression. Tregs may contribute to prevent autoimmune diseases and play a role in transplantation tolerance. Previous studies indicated that the increase of Treg in decidual tissue may be due either to the local expansion or to their selective recruitment at the maternal–fetal interface (9, 78).

Another remarkable cell population present in the decidua is represented by CD14^+^ cells, generally described as decidual myeloid cells (monocytes/macrophages) that did not express CD1a, a dendritic cell (DC) marker (79, 80). Histochemical analysis revealed that they may be in close association with dNK cells, and be involved in functional crosstalks with NK cells. *In vitro* experiments revealed that the interaction between dNK and dCD14^+^ cells results in the production of IFN-γ which, in turn, induces indoleamine 2,3-dioxygenase (IDO) expression in dCD14^+^ cells. Such “conditioned” dCD14^+^ cells acquire the ability to induce Treg by a mechanism that involves TGF-β production. Our perception of the role of dNK cells has thus evolved indeed, not only they do not attack trophoblastic cells but also they can play a major role in immune regulation, by promoting the development of Tregs upon functional interaction with dCD14^+^ cells (81) (Figure 1).

Decidual stromal cells represent other important component of decidual tissue. Indeed, decidual leukocytes are deeply influenced by these cells. On the other hand, also immune cells may modulate DSC function (82). Experimental evidences revealed that DSC, present in the early and late phases of pregnancy, may contribute to the induction of an anti-inflammatory and tolerogenic microenvironment crucial for the establishment/maintenance of successful pregnancy. In this context, DSC have been shown to induce downregulation of major activating NK receptors and to inhibit NK cell proliferation, cytotoxicity, and IFN-γ production. These inhibitory activities are related to the production of PGE2 and to the expression of IDO, resulting in kynurenine production. DSC display another important functional capability related to their ability to promote CD34^+^ cell differentiation. In this context, it has been described that endometrium and decidua contain CD34^+^ cell precursors (dCD34^+^) capable of differentiating *in vitro* into NK cells. Interestingly, even in the absence of exogenous cytokines, dCD34^+^ could give rise to dNK cells when co-cultured with DSC. Other leukocyte functional capabilities may be affected as a result of crosstalk with DSC (Figure 1). Thus, DSC, through IDO and PGE2, may affect the differentiation of PB-CD14^+^ cells toward DCs (82). Moreover, previous studies showed that placenta-derived stromal cells can mediate T cell suppression and induce Treg expansion through a mechanism involving IDO, PGE2, and PD-L1 (83).

Interaction among different cell types may imply a bi-directional crosstalk. Accordingly, also immune cells may influence DSC properties. For example, decidual ILC3 (in particular LTi-like cells) can induce upregulation of ICAM1 and VCAM1 on DSC. This effect is one of the starting events necessary for the development of secondary and tertiary lymphoid organs. In decidua, this effect could be involved in tissue remodeling, placentation, and leukocyte recruitment. Moreover, engagement of RANK has been shown to promote production of CCL2, which, in turn, favors DSC survival and proliferation (84). Moreover, since decidual ILC3 express RANKL it would be of interest to explore whether they can interact with DSC *via* RANKL/RANK-mediated mechanisms.

## Conclusion

As outlined in this contribution, during the first trimester of pregnancy, different cellular players deeply involved in the balance between inflammation and tolerance are present in decidual. In particular, decidual ILC, including NK cells, ILC3 and ILC1, upon interaction with stromal cells, neutrophils, myelomonocytic cells, and T lymphocytes may play a key role in the induction and maintenance of pregnancy. Notably, decidual ILC can originate from CD34^+^ precursors or immature lymphoid cells present *in situ*. In view of the role of ILC in pregnancy, it is possible to speculate that defects in ILC generation or functional interactions in decidual tissues may be a possible cause of fetal losses. While a better knowledge on these cells is clearly required before planning any future clinical application, these concepts open new scenarios of investigation both in innate immunity and in reproductive immunology.

## Author Contributions

All authors discussed together the general outline of the article. PV, CV, and LM wrote the first draft that was subsequently reviewed by MAC, EM, and MCM. Thereafter, all authors contributed to the elaboration of the final version of the manuscript.

## Conflict of Interest Statement

The authors declare that the research was conducted in the absence of any commercial or financial relationships that could be construed as a potential conflict of interest.
